# Factors Associated with Delayed Graft Function in a Universal Hypothermic Machine Perfusion Program: A Single-Center Retrospective Cohort Study

**DOI:** 10.3390/diagnostics16203393

**Published:** 2026-10-19

**Authors:** Andres Calva Lopez, Daniel Sanchez Zalabardo, Sara Esparza Alamanzon, Maria Rodriguez Gomez, Carlos Andres Yanez Ruiz, Vanessa Talavera Cobo, Lucia Polanco Pujol, Francisco Javier Ancizu Marckert, Luis Labairu Huerta, Fernando Jose Diez-Caballero Alonso, Fernando Ramon de Fata Chillon, Bernardino Miñana Lopez

**Affiliations:** 1 Department of Urology, Clínica Universidad de Navarra, 31008 Pamplona, Spain; acalva@unav.es (A.C.L.); dsanchezz@unav.es (D.S.Z.); sesparzaa@unav.es (S.E.A.); mrodriguezgo@unav.es (M.R.G.); cyanezruiz@unav.es (C.A.Y.R.); vtalavera@unav.es (V.T.C.); lpolancopuj@unav.es (L.P.P.); fancizu@unav.es (F.J.A.M.); llabairu@unav.es (L.L.H.); fdcaballero@unav.es (F.J.D.-C.A.); 2 Department of Urology, Clínica Universidad de Navarra, 28027 Madrid, Spain; bminana@unav.es

**Keywords:** kidney transplantation, hypothermic machine perfusion, delayed graft function, diabetes mellitus, hemoglobin

## Abstract

**Background/Objectives**: Hypothermic machine perfusion (HMP) has established itself as an evidence-based and effective preservation strategy to improve graft function and survival after kidney transplantation. It demonstrated a reduction in the incidence of delayed graft function (DGF) compared to static cold storage. However, the risk factors for DGF that persist when HMP is used universally are poorly defined. The aim of this study was to explore donor and recipient factors associated with DGF in a universal HMP kidney transplantation program. **Methods:** A single-center retrospective cohort included 134 consecutive deceased-donor kidney transplants (KTs). The primary endpoint was the incidence of DGF. Secondary endpoints included functional results, complications and survival during follow-up. Univariable and multivariable logistic regression models were performed to identify factors associated with DGF. Graft and patient survival were estimated by the Kaplan–Meier method. Graft function was assessed by serum creatinine (Cr) and estimated glomerular filtration rate (eGFR). **Results:** The overall incidence of DGF was 9.7% (n = 13). When stratified by donor type, the incidence of DGF was 13.3% (n = 10) for donors after brain death (DBD) and 5.1% (n = 3) for donors after circulatory death (DCD). DGF was associated with impaired graft function during follow-up, a longer in-hospital stay and a higher incidence of major complications. Donor diabetes mellitus (DM) (adjusted OR 4.09, 95% CI 1.14–14.62; *p* = 0.030) and lower recipient pre-KT hemoglobin (Hb) (adjusted OR 0.62 per 1 g/dL, 95% CI 0.41–0.93; *p* = 0.021) were associated with DGF; Firth penalized estimates were consistent, but the association with donor DM was attenuated after adjustment for donor age and CIT (OR 3.38, 95% CI 0.91–12.00). Overall graft and patient survival were 99.3% and 100%, respectively, at 12 months, and 99.3% and 96.9%, respectively, at 36 months. **Conclusions:** Lower recipient pre-KT Hb was consistently associated with DGF in this exploratory cohort, whereas the association with donor DM was attenuated after adjustment for donor age and CIT. Within this universal HMP program, DGF incidence did not differ significantly between DBD and DCD grafts; however, in the absence of an SCS comparator, this observation cannot be attributed to HMP. Given the limited number of events (n = 13), the lack of detailed donor DM information and the absence of PRA data, these findings are hypothesis-generating and require validation in larger prospective studies.

## 1. Introduction

Kidney transplantation (KT) is the gold-standard treatment for end-stage kidney disease (ESKD). Kidney graft preservation is essential for adequate graft function after transplantation. Conventional donors, until recent years, were donors after brain death (DBD). However, the increasing demand of waitlisted ESKD patients allowed for the implementation of new donor groups, such as donors after circulatory death (DCD) and expanded criteria donors (ECDs). By widening the donor pool criteria, the quality of kidney grafts has become suboptimal, limiting functional results after KT.

Preservation techniques have primarily relied on hypothermic preservation, reducing graft metabolism and limiting tissue degradation. Traditional graft preservation techniques include static cold storage (SCS), which consists of flushing and submerging the graft in a preservation solution, maintaining a temperature between 0 and 4 °C. Irrespective of the metabolic changes induced by hypothermia, cold ischemia still degrades viable renal tissue, resulting in intracellular acidosis and disruption of the cell cycle, causing apoptosis and necrosis [1,2]. During graft reperfusion, altered molecular cascades during hypothermia become augmented, resulting in ischemia–reperfusion injury (IRI), further deteriorating graft function, with the activation of diverse inflammatory and oxidative pathways [3,4]. In the clinical context, IRI translates to a reduced graft function after KT, particularly delayed graft function (DGF), limiting graft short- and long-term function, resulting in higher morbidity and healthcare costs.

As a means of mitigating the effect of DGF on graft function, hypothermic machine perfusion (HMP) emerged as an alternative to SCS. Multiple trials comparing HMP vs. SCS demonstrated an increased graft survival and reduced incidence of DGF [5,6,7,8], whose results have been confirmed by various systematic reviews and meta-analysis [9,10,11]. Despite the growing evidence in favor of HMP, SCS remains as the default preservation technique in many KT programs worldwide. Although HMP reduces the incidence of DGF compared with SCS, the determinants of DGF in the setting of universal HMP are poorly defined. This study aims to explore the factors associated with DGF in this setting to inform pre-transplant risk assessment and possible interventions.

## 2. Materials and Methods

### 2.1. Study Design

A retrospective single-center cohort study was conducted on all adult kidney transplants from deceased donors (DBD and Maastricht III-only DCD) performed in Clínica Universidad de Navarra in Pamplona, Spain, between January 2022 and December 2024. Recipients from living-donor kidney transplantation or patients receiving multiple-organ transplantation were excluded from the study. Anonymized data were obtained from available donor registries (provided by the National Transplant Organization [ONT] of Spain) and recipient clinical charts. Donor information included demographics, comorbidities, cause of death and pre-procurement laboratory values. All donor data were obtained from the standardized ONT donor registry, which collects specific variables for every deceased donor in Spain. The registry, however, does not have complete data on duration of diabetes mellitus (DM), glycemic control (HbA1c levels), previous antidiabetic therapy, or the presence and/or severity of diabetic nephropathy in donors. Therefore, donor DM was dichotomized based on registry reports of DM as a comorbidity, without further specification.

Data for panel reactive antibodies (PRAs) were not consistently available in our cohort and were therefore not included in the analysis. Our program’s clinical operations initiate the KT procedure once the Department of Nephrology has confirmed the recipient’s immunological compatibility with the graft through crossmatch testing and HLA compatibility evaluation. While PRA levels are routinely measured during pre-KT evaluations, the retrospective nature of this study and limitations of the existing registry data led to inconsistent recording of PRAs; thus, systematic analysis was not possible. PRAs were therefore not included as a variable in our multivariable analysis. PRAs are a known risk factor for DGF and primary non-function (PNF) [12], and its exclusion is an important limitation of the current research.

The primary endpoint was the incidence of DGF, defined as the need for hemodialysis during the first 7 days after transplantation. Secondary endpoints included: incidence of functional delayed graft function (fDGF), defined as the failure of at least a 10% decrease in serum creatinine (Cr) on 3 consecutive days during the first 7 days after transplantation; incidence of PNF, defined as the permanent lack of graft function after transplantation requiring renal replacement therapy (RRT); graft function on postoperative day (POD) 7 at discharge and at 1, 3, 6, 12, 24 and 36 months after transplantation using Cr and estimated glomerular filtration rate (eGFR) as defined by the 2021 Chronic Kidney Disease Epidemiology Collaboration (CKD-EPI) equation; incidence of postoperative complications according to the Clavien–Dindo classification [13]; length of in-hospital stay; graft survival; and patient survival. “Discharge” values were obtained on the day of hospital discharge, which differed between groups; therefore, they do not represent a fixed time point and are shown for descriptive purposes only. Values on POD 7 are shown as a fixed early time point (available in 97 recipients; missing values correspond to recipients without DGF discharged before or on POD 7).

Acute graft rejection (AGR) was defined as biopsy-proven acute rejection according to Banff criteria, or in the absence of renal biopsy, as clinically suspected rejection requiring corticosteroid therapy or plasmapheresis. The course of treatment was determined by the treating nephrologist based on clinical, biochemical and histological evidence. All episodes of AGR were recorded during in-hospital stay and follow-up.

### 2.2. Hypothermic Machine Perfusion Protocol

All kidney grafts were preserved by HMP using the LifePort Kidney Transporter^®^ (Organ Recovery Systems, Itasca, IL, USA), by means of arterial cannulation and perfusion with PumpProtect^®^ (Carnamedica, Warsaw, Poland). Perfusion systolic pressure was set at 30 mmHg, and temperature was maintained below 4 °C. Machine perfusion parameters were recorded and were locally monitored by an assigned circulating nurse. An on-call urologist was available for incidences during machine perfusion.

Two HMP methods were used depending on the logistics of graft procurement. Continuous HMP was initiated immediately after organ procurement in 119 grafts (88.8%). In 15 cases (11.2%), end-ischemic HMP was used, in which the grafts suffered a short period of static cold storage (SCS) in transit from different autonomous communities within the framework of the inter-community exchange program in Spain, and later on, they were switched to HMP upon arrival to our center. Mean supplemental SCS duration for end-ischemic HMP grafts was 1.8 ± 1.0 h. A sensitivity analysis was performed comparing the incidence of DGF between continuous and end-ischemic HMP to evaluate the potential impact of this logistical difference. Baseline characteristics by HMP modality are shown in Supplementary Table S1.

### 2.3. Transplant Protocol

All kidney transplants were performed via a lateral pararectal incision with a conventional extraperitoneal approach. This approach has been the standard surgical technique in our department for over 50 years, with accumulated institutional expertise. The right iliac fossa was the preferred site for all primary kidney transplants, unless contraindicated by anatomical variations or previous surgical interventions. When right-sided access was not feasible, the left iliac fossa was used. The pararectal incision was performed approximately 2–3 cm lateral to the midline, extending from just above the inguinal ligament to a point approximately 5–6 cm above it. The anterior rectus sheath was incised, and the rectus abdominis muscle was retracted medially (or split in select cases) to expose the retroperitoneal space. The peritoneum was mobilized medially to expose the iliac vessels. The external iliac artery and vein were dissected and controlled with vessel loops. Vascular anastomoses were performed using a standardized technique with 6/0 PROLENE™ (Ethicon, Raritan, NJ, USA) continuous sutures. The ureterovesical anastomosis was performed with 5/0 Novosyn^®^ (B. Braun, Melsungen, Germany) suture via a modified Lich-Gregoir extravesical technique (Campos Freire technique), with the insertion of a 4.7 F × 20 cm InLay Optima^®^ ureteral double-J stent (BD, Franklin Lakes, NJ, USA). Foley catheter was removed on postoperative day (POD) 5 and surgical drain was removed on POD 6 unless the recipient’s clinical condition required otherwise. The Department of Nephrology proposed hospital discharge on POD 7 or 8. In some cases, hospital discharge was delayed due to immunosuppressive regimen therapeutic levels. Ureteral stent was removed 1 month after transplantation via flexible cystoscopy in the outpatient Urology clinic. Follow-up after kidney transplantation was coordinated in conjunction with the Department of Nephrology.

### 2.4. Immunosuppressive Regimen

The general immunosuppressive regimen in our program consists of:

(a) Basiliximab 20 mg during transplantation and on POD 4.
(b) Methylprednisolone 5 mg/kg during transplantation; 1 mg/kg on POD 1; reduction by 10 mg per POD until reaching 20 mg/day from POD 4 onwards. Gradual dose reduction should aim for 5 mg/day at 12 weeks, possibly suspending treatment if no AGR occurs.
(c) Tacrolimus 0.14 mg/kg every 24 h from POD 1, targeting a therapeutic range of 8–12 ng/mL during the first 12 weeks, aiming for 6–8 ng/mL onwards.
(d) Mycophenolic acid 720 mg prior to transplantation and every 12 h onwards; reduction to 540 mg or 360 mg every 12 h once Tacrolimus therapeutic range has been achieved.

### 2.5. Statistical Analysis

Qualitative (categorical) variables were expressed as percentages (%) with 95% confidence intervals (CIs) according to Wilson’s method, when applicable. Quantitative (continuous) variables were expressed as mean and standard deviation (SD) or median and interquartile range (IQR). A univariable logistic regression model was performed, reporting crude odds ratio (OR) with 95% CI and *p*-values. Odds ratios for continuous variables are reported per clinically meaningful unit, as indicated in each table (e.g., pre-KT Hb per 1 g/dL); ORs per 1-SD increase are provided in Supplementary Table S5. Candidate donor and recipient variables were defined a priori based on the literature (Table 5). Variables with *p* < 0.05 in the univariable analysis were entered into the multivariable model, and sensitivity analyses were performed post hoc. Because this data-driven selection strategy may overestimate effect sizes when the number of events is small, the multivariable analysis is considered exploratory. As an additional sensitivity analysis, we fitted a model including established risk factors for DGF (donor age and CIT) regardless of their univariable *p*-values. Multivariable models integrated donor and recipient risk factors, reporting adjusted OR with 95% CI and *p*-values.

Secondary continuous endpoints were analyzed with the Mann–Whitney U test or Student’s *t*-test, as appropriate, while categorical endpoints were compared using the Chi-Squared or Fisher’s exact test. Longitudinal graft function (eGFR and Cr) was analyzed with linear mixed-effects models including DGF and time as fixed effects and a random intercept per recipient, which accounts for repeated measurements and unequal follow-up; the DGF × time interaction was tested by a likelihood-ratio test. Comparisons at individual time points are presented as descriptive, with *p*-values adjusted for multiple comparisons using the Holm method. Post hoc sensitivity analyses were performed by including donor type (DCD vs. DBD, reference) or HMP modality (end-ischemic vs. continuous, reference) in the multivariable logistic regression model. Receiver operating characteristic (ROC) curves were generated with area under the curve (AUC) and 95% CI by the DeLong method to assess the discriminative ability of the model and each factor. Optimal cutoff points were identified by the Youden index.

Given the small number of DGF events (n = 13), we adopted a conservative modeling approach to avoid overfitting. The final model yielded an events-per-variable (EPV) ratio of 6.5 (13 events/2 predictors), below the recommended minimum of 10–15 EPV [14,15]. Thus, the findings should be considered as preliminary and hypothesis-generating, and require external validation in larger cohorts. All multivariable models were also fitted using Firth’s penalized-likelihood logistic regression to reduce small-sample bias, with 95% CIs and *p*-values obtained by profile penalized likelihood (Supplementary Table S3) [16]. Conventional maximum-likelihood estimates were retained as the primary analysis because there was no separation in the data, the Firth and maximum-likelihood estimates were very similar, and Firth’s penalization biases predicted probabilities towards 0.5, which would distort the calibration and discrimination analyses [17]. All multivariable results are considered exploratory associations rather than validated independent predictors. We internally validated our model using bootstrap resampling (1000 iterations) and leave-one-out cross-validation (LOOCV) to assess the stability of the estimates. The optimism-adjusted AUC was calculated to give a more proper estimate of the model’s discriminative ability in future populations; because the variable selection step was not repeated within each bootstrap sample, it may underestimate the true optimism. Calibration was evaluated using the Hosmer–Lemeshow goodness-of-fit test, a calibration plot, the bootstrap optimism-corrected calibration slope and the Brier score (Supplementary Table S2; Supplementary Figure S1).

All statistical analyses were performed using SPSS version 30.0 (IBM, Armonk, NY, USA).

## 3. Results

### 3.1. Baseline Characteristics

Baseline characteristics for donors and recipients are shown in Table 1. A total of 134 patients were included in the study. Fifty-six percent of grafts were DBD (n = 75) and 44% were DCD (n = 59), most of which were male donors, and about two-thirds met ECD criteria (n = 86). Median donor age was 59 years (IQR 50–67). The ,most frequent donor comorbidities were history of tobacco consumption, hypertension and DM. The primary cause of death was stroke. Donor kidney function remained preserved at the time of graft procurement with a mean Cr 0.76 ± 0.29 mg/dL and eGFR 98.7 ± 20.6 mL/min/1.73 m^2^.

**Table 1:** Baseline characteristics of donors and recipients ^1^.

	DBD (n = 75)	DCD (n = 59)	
**Donor Characteristics**			
Donor criteria			
ECD	51 (68.0)	35 (59.3)	*p* = 0.365
SCD	24 (32.0)	24 (40.7)	*p* = 0.365
Male sex	37 (49.3)	41 (69.5)	***p *** **= 0.022**
Age (years)	60 (49–69)	59 (52–67)	*p* = 0.537
BMI	26.9 ± 5.3	27.3 ± 4.9	*p* = 0.686
Cause of death			
Stroke	51 (68.0)	17 (28.8)	***p *** **< 0.001**
Anoxia	7 (9.3)	18 (30.5)	***p *** **= 0.003**
Traumatic brain injury	13 (17.3)	9 (15.3)	*p* = 0.817
Other	4 (5.3)	15 (25.4)	***p *** **= 0.001**
Comorbidities			
Hypertension	26 (34.7)	27 (45.8)	*p* = 0.216
DM	10 (13.3)	13 (22.0)	*p* = 0.249
Tobacco	46 (61.3)	39 (66.1)	*p* = 0.593
Pre-procurement labs			
Creatinine	0.80 ± 0.30	0.72 ± 0.28	*p* = 0.086
eGFR	95.9 ± 20.9	102.4 ± 19.9	***p *** **= 0.039**
**HMP**			
CIT (hours)	17.9 ± 4.1	17.6 ± 3.7	*p* = 0.750
HMP time (hours) ^2^	11.3 ± 4.4	11.1 ± 3.9	*p* = 0.836
Perfusion modality			
Continuous	69 (92.0)	50 (84.7)	*p* = 0.270
End-ischemic	6 (8.0)	9 (15.3)	*p* = 0.270
Additional SCS time (hours) ^3^	1.4 ± 0.9	2.0 ± 1.0	*p* = 0.250
**Recipient Characteristics**			
Male sex	51 (68.0)	34 (57.6)	*p* = 0.278
Age (years)	58 (50–68)	60 (47–69)	*p* = 0.971
BMI	26.1 ± 4.4	25.3 ± 4.5	*p* = 0.272
Cause of ESKD			
Diabetic nephropathy	16 (21.3)	10 (16.9)	*p* = 0.661
Glomerulonephritis	18 (24.0)	6 (10.2)	***p *** **= 0.043**
Hepatorenal polycystic disease	10 (13.3)	8 (13.6)	*p* = 1.000
Nephroangiosclerosis	7 (9.3)	9 (15.3)	*p* = 0.422
Other	5 (6.7)	8 (13.6)	*p* = 0.242
Unknown	19 (25.3)	18 (30.5)	*p* = 0.562
Comorbidities			
Hypertension	70 (93.3)	53 (89.8)	*p* = 0.535
DM	23 (30.7)	17 (28.8)	*p* = 0.851
Tobacco	44 (58.7)	30 (50.8)	*p* = 0.387
Previous transplant	12 (16.0)	8 (13.6)	*p* = 0.809
Previous abdominal surgery	45 (60.0)	38 (64.4)	*p* = 0.720
Type RRT			
Hemodialysis	53 (70.7)	40 (67.8)	*p* = 0.850
Peritoneal dialysis	20 (26.7)	13 (22.0)	*p* = 0.553
Time on RRT (months) ^4^	19.5 (11.9–32.5)	21.4 (16.1–31.7)	*p* = 0.516
Pre-KT labs			
Hemoglobin	11.9 ± 1.5	12.3 ± 1.7	*p* = 0.142
Creatinine	6.6 ± 2.7	6.4 ± 2.2	*p* = 0.875
eGFR	9.7 ± 5.1	9.3 ± 4.2	*p* = 0.973
HLA mismatch	5 (4–5)	4 (3–5)	*p* = 0.150
Operative time (minutes)	250 (217–287)	244 (219–278)	*p* = 0.580
In-hospital stay (days)	8 (7–12)	8 (7–10)	*p* = 0.227
Follow-up (months)	24.5 (13.9–36.7)	24.0 (12.8–32.1)	*p* = 0.265

^1^ Values are expressed as n (%), mean ± SD or median (IQR). ^2^ Available in 55 grafts (28 DBD, 27 DCD). ^3^ Applies only to end-ischemic HMP. ^4^ Preemptive transplants excluded (n = 126). Categorical variables were compared with Fisher’s exact test and continuous variables with Student’s *t*-test or the Mann–Whitney U test, according to their distribution.

All kidney grafts were preserved by HMP; 119 grafts were preserved by continuous HMP while the remaining 15 grafts were preserved by end-ischemic HMP as these grafts were procured outside our program’s health district. Mean cold ischemia time (CIT) was 17.8 ± 3.9 h. Mean HMP time (available in 55 grafts) was 11.2 ± 4.1 h.

Median recipient age was 60 years (49–68), most of which were male (n = 85). The most frequent recipient comorbidities were hypertension, history of tobacco consumption and DM. The most frequent cause of ESKD was diabetic nephropathy. The most frequent RRT modality was hemodialysis, with a median time on RRT of 21 months [13,14,15,16,17,18,19,20,21,22,23,24,25,26,27,28,29,30,31,32]; eight recipients were transplanted preemptively. Hospital admission laboratory findings included a mean hemoglobin (Hb) 12.1 ± 1.6 g/dL, mean Cr 6.5 ± 2.5 mg/dL and mean eGFR 9.5 ± 4.7 mL/min/1.73 m^2^. Median operative time was 246 min (218–282).

#### Comparison of HMP Modalities

Compared with continuous HMP (n = 119), the end-ischemic HMP group (n = 15) had a higher proportion of donors with anoxia as cause of death (40.0% vs. 16.0%; *p* = 0.036), a shorter HMP time (8.4 vs. 11.7 h; *p* = 0.032), younger recipients (48 vs. 61 years; *p* = 0.041), a higher proportion of unknown cause of ESKD (53.3% vs. 24.4%; *p* = 0.029) and fewer recipients with previous abdominal surgery (33.3% vs. 65.5%; *p* = 0.023) (Supplementary Table S1). Given the small size of the end-ischemic group, these comparisons are descriptive and should be interpreted with caution. The incidence of DGF was 10.1% (n = 12) in the continuous HMP group and 6.7% (n = 1) in the end-ischemic HMP group (*p* = 1.000), and fDGF rates were 16.0% (n = 19) and 26.7% (n = 4), respectively (*p* = 0.290). After adjustment for donor DM and recipient pre-KT Hb, end-ischemic HMP (reference: continuous HMP) was not associated with DGF (adjusted OR 0.93, 95% CI 0.09–9.21; *p* = 0.950). Given the small size of the end-ischemic group (n = 15, with a single DGF event), this analysis has very limited statistical power and its estimates are imprecise.

### 3.2. Functional Results

Overall, the incidences of DGF and fDGF were 9.7% (n = 13) and 17.2% (n = 23). When stratified by DBD and DCD, the incidence of DGF was 13.3% (n = 10) and 5.1% (n = 3), and the incidence of fDGF was 16.0% (n = 12) and 18.6% (n = 11), respectively. There was only a single case of PNF. There were no statistically significant differences in the incidence of DGF (*p* = 0.145) or fDGF (*p* = 0.818) when stratified by DBD or DCD.

Short- and long-term graft function was impaired in recipients with DGF. On POD 7, mean Cr was 5.81 ± 2.18 vs. 2.52 ± 1.87 mg/dL and mean eGFR 10.8 ± 4.9 vs. 37.8 ± 23.5 mL/min/1.73 m^2^ in the DGF and no-DGF groups, respectively (both *p* < 0.001; data available in 97 recipients). Because recipients with DGF were dialysis-dependent during the first week, creatinine-based eGFR at POD 7 does not reflect true graft function in this group. At discharge, the DGF group presented a mean Cr 3.47 mg/dL and mean eGFR 21.4 mL/min/1.73 m^2^, compared to a mean Cr 1.94 mg/dL and mean eGFR 43.2 mL/min/1.73 m^2^ in the no-DGF group (*p* < 0.001); because discharge occurred later in the DGF group, these values are descriptive. In linear mixed-effects models including all measurements from 1 to 36 months (642 measurements from 134 recipients), DGF was associated with a mean eGFR 13.1 mL/min/1.73 m^2^ lower (95% CI −23.1 to −3.2; *p* = 0.010) and a mean Cr 0.46 mg/dL higher (95% CI 0.13–0.78; *p* = 0.006) than in the no-DGF group, with a significant DGF × time interaction (eGFR *p* = 0.010; Cr *p* < 0.001); results were similar with a random slope for time. In descriptive comparisons at individual time points, Cr was higher in the DGF group at all time points (Holm-adjusted *p* = 0.043), whereas eGFR was lower at discharge, 1 and 3 months (adjusted *p* = 0.001, *p* = 0.029 and *p* = 0.022), but the differences from 6 months onwards were not significant after adjustment (adjusted *p* = 0.068–0.070) (Table 2 and Table 3; Figure 1).

**Table 2:** Graft function by serum creatinine ^2^.

		Total		DGF		No-DGF	*p*-Value	Holm-Adjusted *p*-Value
	n	Creatinine	n	Creatinine	n	Creatinine		
**POD7**	97	2.96 ± 2.21	13	5.81 ± 2.18	84	2.52 ± 1.87	***p *** **< 0.001**	–
**Discharge**	134	2.09 ± 1.10	13	3.47 ± 1.52	121	1.94 ± 0.93	***p *** **< 0.001**	***p *** **< 0.001**
**1 month**	134	1.66 ± 0.69	13	2.32 ± 1.04	121	1.59 ± 0.60	***p *** **= 0.002**	***p *** **= 0.009**
**3 months**	134	1.58 ± 0.65	13	1.94 ± 0.47	121	1.54 ± 0.66	***p *** **= 0.001**	***p *** **= 0.009**
**6 months**	133	1.54 ± 0.55	13	1.94 ± 0.62	120	1.50 ± 0.53	***p *** **= 0.008**	***p *** **= 0.030**
**12 months**	132	1.51 ± 0.49	13	1.77 ± 0.51	119	1.48 ± 0.48	***p *** **= 0.027**	***p *** **= 0.043**
**24 months**	72	1.58 ± 0.51	9	2.01 ± 0.64	63	1.52 ± 0.46	***p *** **= 0.014**	***p *** **= 0.043**
**36 months**	37	1.53 ± 0.48	3	2.25 ± 0.45	34	1.47 ± 0.44	***p *** **= 0.017**	***p *** **= 0.043**

^2^ Values are expressed as mean ± SD. *p*-values from the Mann–Whitney U test; Holm adjustment across the seven time points from discharge to 36 months. “Discharge” values were obtained on the day of hospital discharge, which differed between groups; therefore, they do not represent a fixed time point and are shown for descriptive purposes only. Values on POD 7 are shown as a fixed early time point (available in 97 recipients; missing values correspond to recipients without DGF discharged before or on POD 7); in recipients with DGF, creatinine-based eGFR at POD 7 does not reflect graft function because of dialysis dependence. Values on POD 14 were not available.

**Table 3:** Graft function by eGFR ^3^.

		Total		DGF		No-DGF	*p*-Value	Holm-Adjusted *p*-Value
	**n**	eGFR	n	eGFR	n	eGFR		
**POD7**	97	34.1 ± 23.7	13	10.8 ± 4.9	84	37.8 ± 23.5	***p *** **< 0.001**	–
**Discharge**	134	41.1 ± 22.2	13	21.4 ± 14.1	121	43.2 ± 21.9	***p *** **< 0.001**	***p *** **< 0.001**
**1 month**	134	47.7 ± 19.3	13	34.0 ± 17.0	121	49.2 ± 19.0	***p *** **= 0.006**	***p *** **= 0.029**
**3 months**	134	49.5 ± 17.6	13	37.7 ± 15.7	121	50.8 ± 17.3	***p *** **= 0.004**	***p *** **= 0.022**
**6 months**	133	50.7 ± 19.7	13	40.1 ± 18.9	120	51.8 ± 19.5	***p *** **= 0.035**	*p* = 0.070
**12 months**	132	51.0 ± 19.0	13	42.2 ± 18.2	119	52.0 ± 18.9	***p *** **= 0.041**	*p* = 0.070
**24 months**	72	48.4 ± 19.1	9	35.6 ± 16.1	63	50.2 ± 18.9	***p *** **= 0.017**	*p* = 0.068
**36 months**	37	52.1 ± 22.2	3	30.0 ± 2.0	34	54.1 ± 22.1	***p *** **= 0.021**	*p* = 0.068

^3^ Values are expressed as mean ± SD. *p*-values from the Mann–Whitney U test; Holm adjustment across the seven time points from discharge to 36 months. “Discharge” values were obtained on the day of hospital discharge, which differed between groups; therefore, they do not represent a fixed time point and are shown for descriptive purposes only. Values on POD 7 are shown as a fixed early time point (available in 97 recipients; missing values correspond to recipients without DGF discharged before or on POD 7); in recipients with DGF, creatinine-based eGFR at POD 7 does not reflect graft function because of dialysis dependence. Values on POD 14 were not available.

DGF was associated with a higher incidence of in-hospital complications (61.5% vs. 19.8%; *p* = 0.003), as well as of major complications (Clavien–Dindo ≥ grade III; 30.8% vs. 6.6%; *p* = 0.017). AGR was more frequent in recipients with DGF (30.8% vs. 3.3%; *p* = 0.003); although AGR may affect graft outcomes, graft survival in the DGF group was not affected in our cohort, probably owing to the very low event rate (n = 13).

Length of in-hospital stay was significantly longer in the DGF group (14 days vs. 8 days; *p* < 0.001), which could be explained by the higher incidence of AGR episodes, which require additional immunosuppressive treatment and monitoring, as well as the need for RRT. Long-term complications during follow-up remained, individually, virtually unaffected by the presence of DGF; however, the incidence of multiple complications differed significantly between groups (*p* = 0.003). Overall graft survival at 36 months follow-up was 99.3% because of a single case of PNF in the cohort. Patient survival at 36 months follow-up was 96.9%. Only three patients died during follow-up; causes of death were attributed to myocardial infarction (n = 2) and septic shock (n = 1), all in the no-DGF group. Given the absence of events (graft loss or patient death) in the DGF group, a formal log-rank test was not performed; survival curves stratified by DGF are shown for descriptive purposes only. These findings should be interpreted cautiously due to the limited sample size of the DGF group (n = 13) and the very low overall event rate. Detailed functional results during follow-up and graft/patient survival are shown in Table 2, Table 3 and Table 4 and Figure 2, respectively.

**Table 4:** In-hospital and follow-up complications ^4^.

	Total (n = 134)	DGF (n = 13)	No-DGF (n = 121)	
**Hospital stay**				
In-hospital stay (days)	8 (7–10)	14 (12–16)	8 (7–10)	***p *** **< 0.001**
**In-hospital complications**				
Any complication	32 (23.9)	8 (61.5)	24 (19.8)	***p*** ** = 0.003**
Minor complications (Clavien–Dindo ≤ grade II)	20 (14.9)	4 (30.8)	16 (13.2)	*p* = 0.106
Major complications (Clavien–Dindo ≥ grade III)	12 (9.0)	4 (30.8)	8 (6.6)	***p *** **= 0.017**
Type of complication				
Blood transfusion	16 (11.9)	2 (15.4)	14 (11.6)	*p* = 0.655
Hemorrhage	6 (4.5)	2 (15.4)	4 (3.3)	*p* = 0.105
AGR	8 (6.0)	4 (30.8)	4 (3.3)	***p *** **= 0.003**
Multiple complications *	1 (0.7)	0 (0.0)	1 (0.8)	*p* = 1.000
**Follow-up complications**				
Any complication	52 (38.8)	8 (61.5)	44 (36.4)	*p* = 0.131
Minor complications (Clavien–Dindo ≤ grade II)	21 (15.7)	2 (15.4)	19 (15.7)	*p* = 1.000
Major complications (Clavien–Dindo ≥ grade III)	31 (23.1)	6 (46.2)	25 (20.7)	*p* = 0.075
Type of complication				
Graft pyelonephritis	14 (10.4)	0 (0.0)	14 (11.6)	*p* = 0.360
Wound dehiscence	7 (5.2)	1 (7.7)	6 (5.0)	*p* = 0.519
Lymphocele	7 (5.2)	1 (7.7)	6 (5.0)	*p* = 0.519
Sepsis	4 (3.0)	1 (7.7)	3 (2.5)	*p* = 0.338
Wound infection	4 (3.0)	0 (0.0)	4 (3.3)	*p* = 1.000
Multiple complications *	4 (3.0)	3 (23.1)	1 (0.8)	***p *** **= 0.003**

^4^ Values are expressed as n (%) or median (IQR). * Multiple complications is defined as presenting more than one of the listed complications during in-hospital stay or follow-up. Categorical variables were compared with Fisher’s exact test and in-hospital stay with the Mann–Whitney U test.

### 3.3. Factors Associated with Delayed Graft Function

A univariable logistic regression model was employed to identify donor and recipient factors associated with DGF. The donor univariable model revealed DM as the only risk factor significantly associated with DGF (OR 5.24, 95% CI 1.57–17.50; *p* = 0.007). The recipient univariable model revealed higher pre-KT Hb as the only factor significantly associated with a lower risk of DGF (OR 0.57 per 1 g/dL, 95% CI 0.38–0.86; *p* = 0.007). There were no other donor or recipient-related characteristics, or HMP parameters, associated with the risk of DGF. Multivariable logistic regression was performed, in which donor DM (adjusted OR 4.09, 95% CI 1.14–14.62; *p* = 0.030) and recipient pre-KT Hb (adjusted OR 0.62 per 1 g/dL, 95% CI 0.41–0.93; *p* = 0.021) remained associated with DGF (Table 5). Firth penalized logistic regression yielded consistent estimates for donor DM (OR 4.04, 95% CI 1.15–13.71; *p* = 0.031) and pre-KT Hb (OR 0.64 per 1 g/dL, 95% CI 0.42–0.93; *p* = 0.019) (Supplementary Table S3). The discriminative capacity of the multivariable logistic regression model was evaluated by ROC curves and showed a raw AUC of 0.745 (95% CI 0.576–0.915; *p* = 0.005), with a sensitivity of 69% and specificity of 77% at the optimal threshold. Following bootstrap internal validation (1000 iterations), the optimism-adjusted AUC was 0.737, suggesting limited overfitting, although these findings should be interpreted cautiously given the low number of events. The Hosmer–Lemeshow test did not indicate lack of fit (χ^2^ = 11.17, df = 8, *p* = 0.192); however, this test has very limited power with only 13 events. The calibration plot (Supplementary Figure S1) showed close agreement between predicted and observed risks across quintiles of predicted probability; the calibration slope after bootstrap optimism correction was 0.91 (heuristic shrinkage factor 0.84), and the Brier score was 0.073 (0.079 after optimism correction; 0.088 for a null model). Given the small number of events, these estimates are imprecise and should be interpreted with caution.

The discriminative ability of pre-KT Hb alone (AUC 0.733) was nearly equivalent to that of the combined model (AUC 0.745), suggesting that pre-KT Hb is the dominant contributive factor to the model’s discriminative performance, in contrast with the poor discriminative ability of donor DM (AUC 0.661). This likely reflects the low prevalence of donor DM in our cohort, limiting its individual discriminative capacity despite its association with DGF. These findings position pre-KT Hb as the most informative factor in our cohort (Figure 3). The optimal threshold for pre-KT Hb according to the Youden index was ≤10.7 g/dL (sensitivity 54%, specificity 87%). Recipients with pre-KT Hb ≤ 10.7 g/dL had a DGF incidence of 30.4% (7/23) compared with 5.4% (6/111) in recipients above this threshold, corresponding to an absolute risk difference of 25% and a risk ratio of 5.6 (*p* = 0.002). Because this cutoff was derived and evaluated in the same small cohort, these estimates are likely optimistic; the threshold is exploratory and should not be used for clinical decision-making without external validation.

Data for PRA were not always available in the donor registry or in the clinical records of recipients. Therefore, PRA could not be evaluated as a potential factor associated with DGF or included as a variable in the multivariable analysis. This is an important limitation that impedes the assessment of immunologic risk as a confounder in our study.

**Table 5:** Univariable and multivariable logistic regression models for donor and recipient factors associated with DGF.

	Univariable Logistic Regression Model ^5^			Multivariable Logistic Regression Model		
**Donor Factors**	**OR**	**95% CI**	***p *** **-value**	**OR**	**95% CI**	***p *** **-value**
Donor type (DCD vs. DBD)	0.35	0.09–1.33	*p* = 0.122	—	—	—
Male sex	2.60	0.68–9.91	*p* = 0.162	—	—	—
Age (per 10 years)	1.39	0.85–2.27	*p* = 0.187	—	—	—
BMI (per 1 kg/m^2^)	1.01	0.90–1.13	*p* = 0.851	—	—	—
Hypertension	0.95	0.29–3.08	*p* = 0.933	—	—	—
DM	**5.24**	**1.57–17.50**	***p *** **= 0.007**	**4.09**	**1.14–14.62**	***p *** **= 0.030**
Tobacco	1.33	0.39–4.58	*p* = 0.649	—	—	—
Cr (per 0.1 mg/dL)	1.07	0.91–1.27	*p* = 0.409	—	—	—
eGFR (per 10 mL/min/1.73 m^2^)	0.90	0.69–1.16	*p* = 0.414	—	—	—
CIT (per 1 h)	1.07	0.92–1.23	*p* = 0.381	—	—	—
HMP time (per 1 h)	1.13	0.88–1.46	*p* = 0.338	—	—	—
**Recipient factors**	**OR**	**95% CI**	***p *** **-value**	**OR**	**95% CI**	***p *** **-value**
Age (per 10 years)	1.05	0.71–1.56	*p* = 0.809	—	—	—
Male sex	1.33	0.39–4.56	*p* = 0.649	—	—	—
BMI (per 1 kg/m^2^)	0.95	0.83–1.08	*p* = 0.431	—	—	—
Hypertension	1.08	0.13–9.19	*p* = 0.943	—	—	—
DM	2.19	0.69–7.00	*p* = 0.185	—	—	—
Tobacco	1.33	0.41–4.31	*p* = 0.631	—	—	—
Time on RRT (per 12 months)	1.27	0.95–1.70	*p* = 0.101	—	—	—
Hb (per 1 g/dL)	**0.57**	**0.38–0.86**	***p *** **= 0.007**	**0.62**	**0.41–0.93**	***p *** **= 0.021**
Operative time (per 30 min)	1.15	0.85–1.56	*p* = 0.359	—	—	—

^5^ PRA was not included due to incomplete data availability in the cohort; this precludes assessment of PRA as a potential confounder or independent predictor of DGF. ORs for continuous variables are expressed per the unit indicated in each row (ORs per 1-SD increase in Supplementary Table S5). HMP time was available in 55 grafts and time on dialysis in 126 recipients. Firth penalized estimates of the multivariable model are shown in Supplementary Table S3.

A post hoc sensitivity analysis was performed by adding donor type (DCD vs. DBD, reference) to the multivariable model. Donor type was not significantly associated with DGF either in the univariable analysis (OR 0.35, 95% CI 0.09–1.33; *p* = 0.122) or after adjustment for donor DM and recipient pre-KT Hb (adjusted OR 0.28, 95% CI 0.07–1.24; *p* = 0.094). Given the very small number of events, particularly in DCD recipients (n = 3), these estimates are imprecise. The associations of donor DM and pre-KT Hb with DGF were similar in direction and magnitude in this model (Table 6).

A secondary post hoc sensitivity analysis was performed to evaluate the potential confounding effect of HMP modality on DGF by including HMP modality (end-ischemic vs. continuous, reference) in the multivariable logistic regression model along with donor DM and recipient pre-KT Hb; end-ischemic HMP was not associated with DGF (adjusted OR 0.93, 95% CI 0.09–9.21; *p* = 0.950). The present analysis should be considered preliminary due to the small sample size of the end-ischemic HMP cohort (n = 15, with a single DGF event) (Table 7). In a third sensitivity analysis including established risk factors for DGF regardless of their univariable *p*-values (donor age and CIT), fitted with Firth regression, the association with pre-KT Hb was unchanged (OR 0.64 per 1 g/dL, 95% CI 0.42–0.94; *p* = 0.021), whereas the estimate for donor DM was attenuated and no longer statistically significant (OR 3.38, 95% CI 0.91–12.00; *p* = 0.067); donor age (OR 1.10 per 10 years, 95% CI 0.71–1.86) and CIT (OR 1.06 per hour, 95% CI 0.91–1.25) were not associated with DGF (Supplementary Table S3).

To assess internal stability of the main multivariable model, leave-one-out cross-validation (LOOCV) and bootstrap resampling (1000 iterations) were performed. LOOCV showed that the stability of the primary predictors of DGF, donor DM and recipient pre-KT Hb maintained their direction and magnitude consistently across all iterations, with mean OR virtually identical to those of the main model (donor DM OR 4.09; pre-KT Hb OR per 1-SD increase, equivalent to 0.62 per 1 g/dL). Bootstrap analysis showed donor DM with the expected direction in 97.6% of samples (bootstrap 95% CI 1.07–17.8) and pre-KT Hb in 99.4% of samples (bootstrap 95% CI 0.17–0.90 per 1-SD increase). Bootstrap resampling and LOOCV showed that the direction of the associations for donor DM and pre-KT Hb was stable across resamples; however, these procedures do not compensate for the limited information contained in 13 events, and the estimates remain imprecise.

## 4. Discussion

The overall incidence of DGF in our cohort was 9.7%, which is lower than the 20–30% reported in larger series [5,18,19,20,21]. Within this HMP-only cohort, the incidence of DGF did not differ significantly between DBD and DCD grafts (13.3% vs. 5.1%; *p* = 0.145). However, because our study lacks an SCS comparator and includes only 13 DGF events, this finding should not be interpreted as evidence of equivalence, nor can the absence of a difference be attributed to HMP. It is nonetheless consistent with previous data suggesting that machine perfusion reduces DGF irrespective of donor type [8], and it warrants evaluation in comparative studies.

In our cohort there were two methods of HMP: continuous HMP, immediately after procurement (88.8%), and end-ischemic HMP, starting after a short period of SCS during transport (11.2%). Although end-ischemic HMP has been associated with potentially worse outcomes than continuous HMP, the additional SCS time in our cohort was short (1.8 ± 1.0 h), and we did not observe a significant difference in the incidence of DGF between modalities (10.1% vs. 6.7%; *p* = 1.000). This is in line with previous studies suggesting that short periods of SCS prior to HMP may be tolerated [22]. However, the small number of end-ischemic HMP grafts (n = 15) did not provide sufficient power to detect modest differences, and our results cannot be extrapolated to longer periods of SCS before HMP. In clinical practice, if HMP is not readily available at the procurement center, a short CIT by SCS may be acceptable if HMP is initiated promptly upon arrival at the implanting center.

Donor DM and lower recipient pre-KT Hb were associated with DGF. The association with pre-KT Hb was consistent in magnitude and statistically significant in all models. Donor DM was associated with approximately 4-fold higher odds of DGF, an estimate that was similar to Firth regression after adjustment for donor type. After additional adjustment for donor age and CIT, the estimate was attenuated (OR 3.38, 95% CI 0.91–12.00; *p* = 0.067), mainly owing to donor age (OR 3.52 when only donor age was added), as diabetic donors were older (median 62 vs. 59 years) and more frequently met ECD criteria (83% vs. 60%). The OR therefore remained of a similar order of magnitude, and the loss of statistical significance is compatible both with partial confounding by donor age and with the limited power of a four-variable model with only 13 events (EPV 3.3); nevertheless, it indicates that the association with donor DM may be partly explained by other donor characteristics and should be interpreted with particular caution. This is biologically plausible, as persistent hyperglycemia and microvascular damage in diabetic donors may impair the ability of the kidney to resist cold ischemia and reperfusion injury, resulting in a diminished functional recovery after KT. There is empirical evidence that increased oxidative stress, endothelial dysfunction and mitochondrial dysfunction are associated with diabetic nephropathy and may predispose grafts to IRI [23,24,25,26].

Interestingly, these results are in contrast with much of the published literature, where recipient DM is a well-established independent risk factor for DGF [27,28,29,30]. This has been corroborated in recent systematic reviews and meta-analyses, where DM increases the risk of DGF by 52–80% in both deceased- and living-donor KT [31,32]. Donor DM was categorized in a binary registry variable with no information on duration, glycemic control, treatment modalities or diabetic nephropathy, which could misclassify donors and bias the relationship with DGF in either direction. Diabetic donors are more often classified as ECDs and have other comorbidities which could cause residual confounding. The absence of data on diabetic nephropathy is particularly limiting, as it prevents the distinction between donors with normal renal histology and those with established DM-related kidney impairment. Therefore, our finding should be viewed as hypothesis-generating, rather than conclusive. Additional studies with complete donor DM characterization are needed to determine if donor DM is truly associated with DGF and to define which aspects of donor DM (duration, treatment or nephropathy) are of greatest clinical importance. But until then, graft selection or allocation of diabetic donors should be done cautiously.

The role of recipient anemia as a determinant factor in graft function has been less frequently assessed compared to other donor and recipient characteristics; nevertheless, the biological rationale is compelling. Patients with ESKD present anemia due to erythropoietin (EPO) deficiency and RRT-associated blood loss, especially with hemodialysis. Kidney graft hypoxia after reperfusion is associated with IRI and Hb is an essential factor for tissue oxygen delivery. Reduced pre-KT Hb might play a role in a lack of adequate oxygen delivery, inducing IRI and evolving to DGF [29]. Thus, oxygenated kidney graft preservation rises as an alternative to mitigate the effect of IRI [33]. However, some studies argue that high pre-KT Hb can increase the risk of graft thrombosis, asserting a U-shaped effect on the incidence of DGF [29], although evidence is very limited. Pre-KT Hb is universally available, inexpensive and potentially modifiable; however, the cutoff identified in our cohort is exploratory, and whether pre-KT Hb optimization reduces the incidence of DGF remains unknown and should be tested in dedicated prospective studies. It could potentially be modified with EPO-stimulating agents and iron supplementation, or in very selected cases, blood transfusion, prior to KT.

The combined multivariable model showed acceptable discriminative ability for KT recipients at risk of developing DGF. Interestingly, pre-KT Hb alone had similar discriminative ability, while donor DM alone had poor discriminative ability. These results reflect the very limited individual discriminative capacity of a low-prevalence variable, with DM present in only 17% of donors, despite its association with DGF, and position pre-KT Hb as the most informative factor in our cohort.

PRA was not included in our analysis because of incomplete retrospective data, not because it is not clinically relevant. PRAs are a well-established risk factor for DGF in the literature [12]. All KTs were performed after crossmatch and HLA compatibility had been confirmed. PRAs are a continuous risk factor, and even low-level sensitization may increase the risk of DGF. We cannot adjust for this confounder due to a lack of PRA data, and our estimates may be biased. In particular, the association of donor DM with DGF may partly be due to PRAs if sensitized recipients (higher PRA levels) were more likely to receive grafts from diabetic donors. Similarly, the protective association between pre-KT Hb and DGF may be confounded if PRA level is correlated with Hb (e.g., longer hemodialysis). We adjusted for available variables, but residual confounding cannot be excluded. We sought to identify factors associated with DGF in the face of ubiquitous HMP, particularly modifiable factors such as Hb. Our findings are not invalidated by the lack of PRA data, but this suggests an incomplete model; the contributions of donor DM and pre-KT Hb require confirmation in studies with complete immunologic data.

The use of a pararectal incision in all KTs is a surgical practice peculiar to our center and may limit the generalizability of our results. This approach is routinely used in our center and is associated with acceptable complication rates. However, other incisions (e.g., Gibson or inguinal) are used by several transplant centers worldwide. A systematic review and meta-analysis found no significant difference in the overall wound complication rate between pararectal and inguinal incisions [34]. The choice of incision may affect surgical outcomes such as wound healing, pain and cosmetic appearance, but is unlikely to have a significant impact on DGF.

Graft and patient survival results were excellent. One-year graft and patient survival were 99.3% and 100% respectively. At 36 months follow-up, overall graft survival remained virtually unaltered at 99.3% while patient survival was 96.9%. These results align favorably with larger HMP cohorts, with similar but slightly lower graft and patient survival rates [5,6,35]. No graft or patient losses occurred in the DGF group, possibly due to the very low incidence of DGF in our cohort, although this cannot be interpreted as evidence of the absence of an effect of DGF on survival. However, KT recipients in the DGF group presented impaired graft function during follow-up, with a mean eGFR 13.1 mL/min/1.73 m^2^ lower than that of the no-DGF group in the mixed-effects model, although differences at individual time points from 6 months onwards were not significant after adjustment for multiple comparisons. This dissociation between survival and functional results in our cohort highlights the importance of DGF-prevention strategies, not only for immediate and short-term results, which include longer in-hospital stays and a higher incidence of major complications, but also for long-lasting consequences on graft function.

There are several limitations to consider. First, the retrospective design may introduce potential selection bias and limits our ability to infer causality, and the absence of an SCS comparator precludes any conclusion on the effect of HMP. Second, the very low number of DGF events (n = 13) poses major limitations on the statistical strength and complexity of our multivariable model, and the data-driven selection of variables may overestimate effect sizes, as discussed above. Third, the number of recipients with 24- and 36-month data was limited (72 and 37, respectively), mainly because of administrative censoring. Although recipients with and without 24-month measurements had comparable baseline characteristics and 12-month graft function, attrition bias cannot be excluded, and the multiple comparisons across time points increase the risk of type I error; long-term functional results should therefore be interpreted with caution. Fourth, while our results suggest pre-KT Hb as a potentially modifiable factor associated with DGF, the benefit of systematic optimization of anemia in KT recipients requires prospective evaluation in dedicated trials. Fifth, PRA data were not consistently available in our cohort and therefore could not be included in the analysis, the exclusion of which constitutes a major unmeasured confounder in our study. Sixth, donor DM was restricted to a binary variable and did not include information on duration, glycemic control, treatment modalities, or diabetic nephropathy. The lack of information may lead to misclassification of severity, and it is difficult to distinguish a well-controlled early DM from a long-standing poorly controlled DM with established diabetic nephropathy, which carries very different risks. Seventh, all episodes of AGR were recorded during in-hospital stay and follow-up; however, the absence of universal biopsy confirmation may have introduced a misclassification bias that could have either over- or underestimated the true incidence of AGR in our cohort. Further studies with detailed DM profiling of the donor are needed to show the real correlation between donor DM and DGF.

Future prospective studies should include systematic PRA collection to confirm the associations of donor DM and pre-KT Hb with the risk of DGF, after adjusting for immunologic variables. Interventional studies are needed to assess whether optimizing Hb levels prior to KT decreases the incidence of DGF stratified by PRA status to assess for potential effect modification. It is important to validate our findings in large, multicenter populations with adequate statistical power and extensive variable adjustment, including PRA, CIT and other well-established risk factors for DGF. These approaches will help elucidate the role of modifiable variables and enhance risk-stratification within the framework of universal HMP.

## 5. Conclusions

In this single-center cohort with universal HMP, the incidence of DGF was low, and did not differ significantly between DBD and DCD grafts, along with excellent graft and patient survival. Because there was no SCS comparator, these observations cannot be attributed only to HMP. Lower recipient pre-KT Hb was consistently associated with DGF across all models, whereas the effect of donor DM was attenuated after adjustment for donor age and CIT. Given the very low incidence of DGF (n = 13), the limited characterization of donor DM and the absence of PRA data, these associations should be considered exploratory and hypothesis-generating. Pre-KT Hb is a potentially modifiable factor, but whether its optimization reduces the risk of DGF should be tested in larger prospective studies with comprehensive immunologic data and external validation.

## Figures and Tables

**Figure 1:**
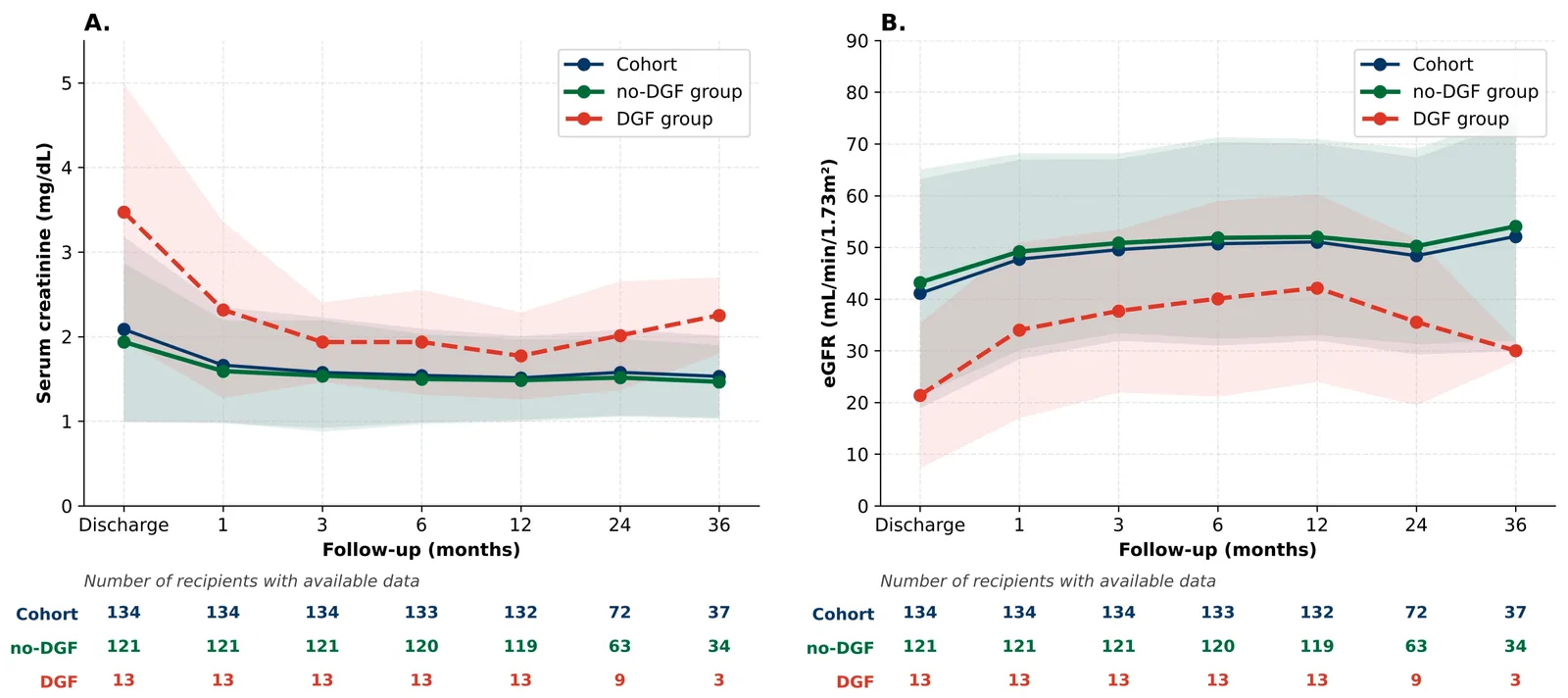
Graft function during follow-up by serum creatinine (Panel (**A**)) and eGFR (Panel (**B**)) in the overall cohort and in the DGF and no-DGF groups. The first time point corresponds to hospital discharge. Lines represent mean values and shaded areas ± SD. Recipients with DGF showed higher Cr and lower eGFR than the no-DGF group throughout follow-up. The number of recipients with available data at each time point is shown below each panel.

**Figure 2:**
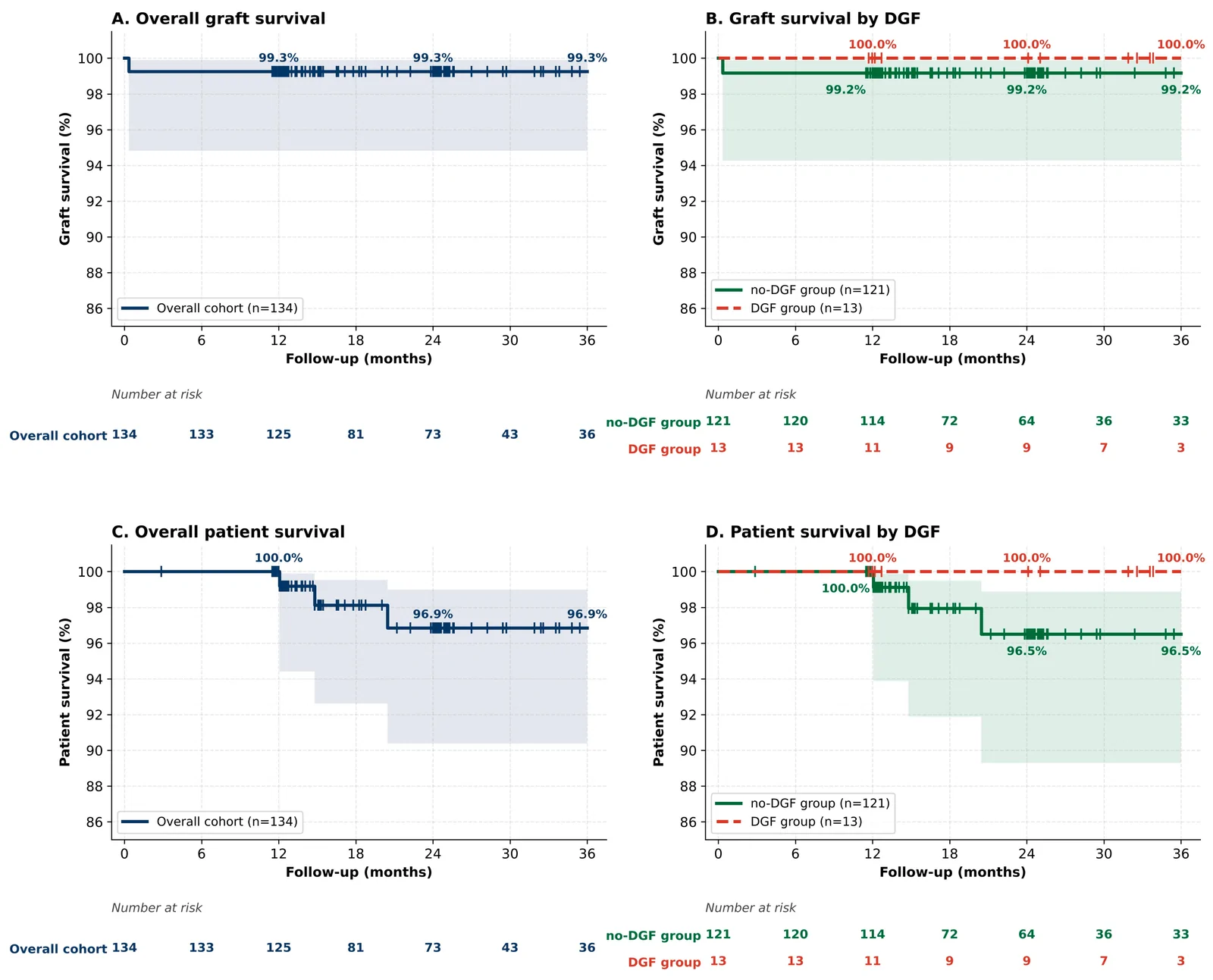
Kaplan–Meier estimates of overall graft survival (Panel (**A**)), graft survival by DGF status (Panel (**B**)), overall patient survival (Panel (**C**)) and patient survival by DGF status (Panel (**D**)). Shaded areas represent 95% CI, tick marks indicate censored observations, and the number of recipients at risk is shown below each panel. Given the very low number of events (one graft loss and three deaths, none in the DGF group), no formal comparison by DGF status was performed, and no comparative inference should be drawn from Panels (**B**,**D**).

**Figure 3:**
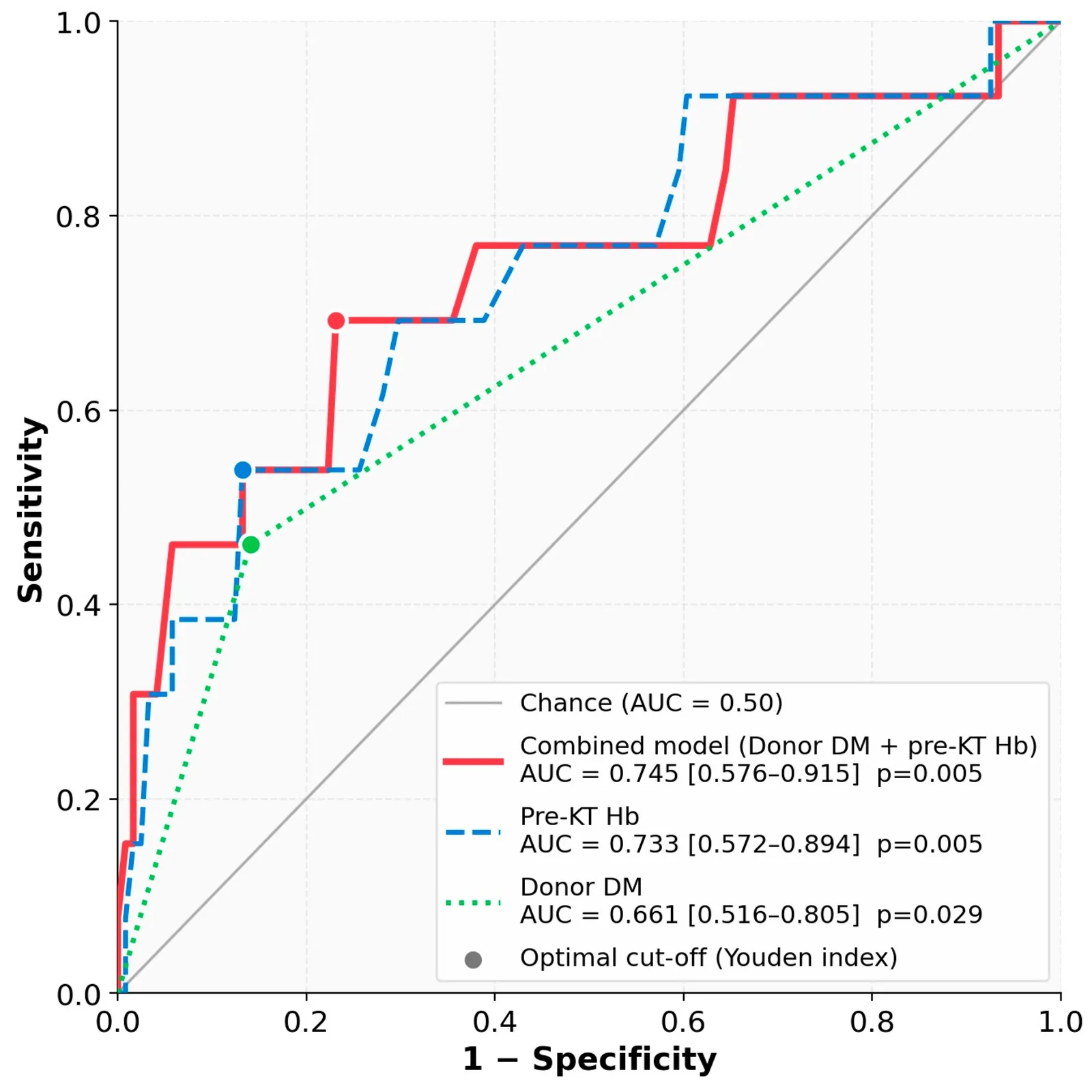
ROC curves for the multivariable model (donor DM and pre-KT Hb) and for each variable alone. Dots indicate the optimal cutoff according to the Youden index.

**Table 6:** Multivariable logistic regression model: Main model and sensitivity analysis (donor type) ^6^.

	Univariable Model OR (95% CI)	*p*-Value	Main model Adjusted OR (95% CI)	*p*-Value	Sensitivity Model Adjusted OR (95% CI)	*p*-Value
Donor DM	**5.24** (1.57–17.50)	***p *** **= 0.007**	**4.09** (1.14–14.62)	***p *** **= 0.030**	**5.31** (1.37–20.52)	***p *** **= 0.016**
Recipient pre-KT Hb (per 1 g/dL)	**0.57** (0.38–0.86)	***p *** **= 0.007**	**0.62** (0.41–0.93)	***p *** **= 0.021**	**0.64** (0.42–0.97)	***p *** **= 0.036**
Donor type (DCD vs. DBD, reference)	0.35 (0.09–1.33)	*p* = 0.122	—	—	0.28 (0.07–1.24)	*p* = 0.094

^6^ Main model: Donor DM and recipient pre-KT Hb. Sensitivity model (post hoc): Main model plus donor type. Firth penalized estimates are shown in Supplementary Table S3.

**Table 7:** Multivariable logistic regression model: Main model and sensitivity analysis (HMP modality) ^7^.

	Univariable Model OR (95% CI)	*p*-Value	Main Model Adjusted OR (95% CI)	*p*-Value	Sensitivity Model Adjusted OR (95% CI)	*p*-Value
Donor DM	**5.24** (1.57–17.50)	***p *** **= 0.007**	**4.09** (1.14–14.62)	***p *** **= 0.030**	3.38 (0.91–12.00)	*p* = 0.067
Recipient pre-KT Hb (per 1 g/dL)	**0.57** (0.38–0.86)	***p *** **= 0.007**	**0.62** (0.41–0.93)	***p *** **= 0.021**	**0.64** (0.42–0.94)	***p *** **= 0.021**
HMP modality (end-ischemic vs. continuous, reference)	—	—	—	—	0.93 (0.09–9.21)	*p* = 0.950

^7^ Main model: Donor DM and recipient pre-KT Hb. Sensitivity model (post hoc): Main model plus HMP modality. Only one DGF event occurred in the end-ischemic group (n = 15). Firth penalized estimates are shown in Supplementary Table S3.

## Data Availability

The datasets presented in this article are not readily available because the data are part of an ongoing study. Requests to access the dataset should be directed to A.C.L. (acalva@unav.es).

## Supplementary Materials

The following supporting information can be downloaded at: https://www.mdpi.com/article/10.3390/diagnostics16203393/s1, Table S1: Baseline characteristics of donors and recipients by HMP modality; Table S2: Multivariable model internal validation; Table S3: Multivariable logistic regression models for DGF: conventional maximum-likelihood and Firth penalized-likelihood estimates; Table S4: Characteristics of recipients with and without Cr and eGFR measurement at 24 months; Table S5: Univariable logistic regression for DGF: odds ratios per clinical unit and per 1-SD increase; Figure S1: Calibration plot of the multivariable model for DGF (donor DM and pre-KT Hb).

## Abbreviations

The following abbreviations are used in this manuscript:

AGR	Acute graft rejection
AUC	Area under the curve
BMI	Body mass index
CI	Confidence intervals
CIT	Cold ischemia time
CKD-EPI	Chronic Kidney Disease Epidemiology Collaboration
Cr	Serum creatinine
DBD	Donor after brain death
DCD	Donor after circulatory death
DGF	Delayed graft function
ECD	Expanded criteria donor
eGFR	Estimated glomerular filtration rate
EPO	Erythropoietin
EPV	Events-per-variable
ESKD	End-stage kidney disease
fDGF	Functional delayed graft function
Hb	Hemoglobin
HLA	Human leukocyte antigen
HMP	Hypothermic machine perfusion
IQR	Interquartile range
IRI	Ischemia–reperfusion injury
KT	Kidney transplantation
LOOCV	Leave-one-out cross-validation
OR	Odds ratio
PNF	Primary non-function
POD	Postoperative day
PRAs ROC	Panel reactive antibodies Receiver operating characteristic
SCS	Static cold storage
SD	Standard deviation
