# Supplementary material for: Factors Associated with Delayed Graft Function in a Universal Hypothermic Machine Perfusion Program: A Single-Center Retrospective Cohort Study
Source: Diagnostics (Basel). 2026 Oct 19;16(20):3393. doi: 10.3390/diagnostics16203393 (PMC13703307; doi:10.3390/diagnostics16203393)
Supplement: Supplementary file 1 [file diagnostics-16-03393-s001.zip › diagnostics-4596111-supplementary.pdf]

Table S1. Baseline characteristics of donors and recipients by HMP modality.

	Continuous HMP (n=119)	End-ischemic HMP (n=15)	
**Donor Characteristics**			
Donor type			
DBD	69 (58.0)	6 (40.0)	p=0.270
DCD	50 (42.0)	9 (60.0)	p=0.270
Donor criteria			
ECD	79 (66.4)	7 (46.7)	p=0.158
SCD	40 (33.6)	8 (53.3)	p=0.158
Male sex	67 (56.3)	11 (73.3)	p=0.271
Age (years)	60 (52-68)	54 (51-64)	p=0.268
BMI	27.1 ± 5.1	27.3 ± 5.2	p=0.896
Cause of death			
Stroke	63 (52.9)	5 (33.3)	p=0.179
Anoxia	19 (16.0)	6 (40.0)	**p=0.036**
Traumatic brain injury	21 (17.6)	1 (6.7)	p=0.464
Other	16 (13.4)	3 (20.0)	p=0.447
Comorbidities			
Hypertension	49 (41.2)	4 (26.7)	p=0.402
DM	22 (18.5)	1 (6.7)	p=0.467
Tobacco	75 (63.0)	10 (66.7)	p=1.000
Pre-procurement labs			
Creatinine	0.77 ± 0.30	0.71 ± 0.28	p=0.463
eGFR	98.0 ± 20.7	104.9 ± 19.9	p=0.224
HMP			
CIT (hours)	17.7 ± 4.1	18.5 ± 2.9	p=0.419
HMP time (hours)[2]	11.7 ± 4.1	8.4 ± 2.8	**p=0.032**
**Recipient Characteristics**			
Male sex	78 (65.5)	7 (46.7)	p=0.166
Age (years)	61 (51-69)	48 (37-61)	**p=0.041**
BMI	26.0 ± 4.4	24.0 ± 4.6	p=0.101
Cause of ESKD			
Diabetic nephropathy	25 (21.0)	1 (6.7)	p=0.301
Glomerulonephritis	21 (17.6)	3 (20.0)	p=0.732
Hepatorenal polycystic disease	17 (14.3)	1 (6.7)	p=0.692
Nephroangiosclerosis	15 (12.6)	1 (6.7)	p=1.000
Other	12 (10.1)	1 (6.7)	p=1.000
Unknown	29 (24.4)	8 (53.3)	**p=0.029**
Comorbidities			
Hypertension	109 (91.6)	14 (93.3)	p=1.000
DM	38 (31.9)	2 (13.3)	p=0.230
Tobacco	66 (55.5)	8 (53.3)	p=1.000
Previous transplant	19 (16.0)	1 (6.7)	p=0.469
Previous abdominal surgery	78 (65.5)	5 (33.3)	**p=0.023**
Type RRT			
Hemodialysis	84 (70.6)	9 (60.0)	p=0.390
Peritoneal dialysis	29 (24.4)	4 (26.7)	p=1.000
Pre-emptive KT	6 (5.0)	2 (13.3)	p=0.220
Time on RRT	20.9 (12.7-32.5)	16.6 (11.5-20.8)	p=0.245
Pre-KT labs			
Hemoglobin	12.0 ± 1.5	12.7 ± 2.3	p=0.131

Creatinine	6.4 ± 2.5	6.9 ± 2.3	p=0.486
eGFR	9.7 ± 4.8	8.4 ± 3.4	p=0.330
HLA mismatch	4 (4-5)	4 (4-5)	p=0.740
Operative time (minutes)	243 (217-279)	270 (234-302)	p=0.109
In-hospital stay (days)	8 (7-11)	7 (6-11)	p=0.174
Follow-up (months)	24.3 (13.4-36.4)	15.5 (12.4-25.1)	p=0.232

Values are expressed as n (%), mean ± SD or median (IQR). [2]Available in 55 grafts (47 continuous, 8 end-ischemic). Categorical variables were compared with Fisher's exact test and continuous variables with Student's t-test or the Mann–Whitney U test, as appropriate.

Table S2. Multivariable model internal validation.

	**Value**
Number of DGF events (n)	13
Number of predictors in final model (n)	2
EPV ratio (n)	6.5
Model AUC (95% CI)	0.745 (0.576-0.915)
Optimism-adjusted AUC (bootstrap)	0.737
Bootstrap samples with the expected direction of effect	
Donor DM (%)	97.6
Pre-KT Hb (%)	99.4
Hosmer–Lemeshow test, χ^2 (df); p-value	11.17 (8); 0.192
Calibration slope, bootstrap optimism-corrected (heuristic shrinkage factor)	0.91 (0.84)
Brier score, apparent / optimism-corrected (null model)	0.073 / 0.079 (0.088)

Table S3. Multivariable logistic regression models for DGF: conventional maximum-likelihood and Firth penalized-likelihood estimates.

	**Maximum likelihood OR (95% CI)**	**p-value**	**Firth penalized likelihood OR (95% CI)**	**p-value**
**Main model**				
Donor DM	**4.09** (1.14–14.62)	**p=0.030**	**4.04** (1.15–13.71)	**p=0.031**
Pre-KT Hb (per 1 g/dL)	**0.62** (0.41–0.93)	**p=0.021**	**0.64** (0.42–0.93)	**p=0.019**
**Sensitivity model 1: donor type**				
Donor DM	**5.31** (1.37–20.52)	**p=0.016**	**4.99** (1.35–18.45)	**p=0.017**
Pre-KT Hb (per 1 g/dL)	**0.64** (0.42–0.97)	**p=0.036**	**0.66** (0.43–0.97)	**p=0.035**
DCD vs DBD (reference)	0.28 (0.07–1.24)	p=0.094	0.33 (0.07–1.20)	p=0.095
**Sensitivity model 2: HMP modality**				
Donor DM	**4.05** (1.10–14.95)	**p=0.036**	**4.07** (1.13–14.20)	**p=0.033**
Pre-KT Hb (per 1 g/dL)	**0.62** (0.41–0.93)	**p=0.022**	**0.65** (0.43–0.94)	**p=0.020**
End-ischemic vs continuous HMP (reference)	0.93 (0.09–9.21)	p=0.950	1.35 (0.12–7.43)	p=0.767
**Sensitivity model 3: established risk factors**				
Donor DM	3.53 (0.93–13.44)	p=0.065	3.38 (0.91–12.00)	p=0.067
Pre-KT Hb (per 1 g/dL)	**0.62** (0.41–0.94)	**p=0.023**	**0.64** (0.42–0.94)	**p=0.021**
Donor age (per 10 years)	1.15 (0.69–1.89)	p=0.598	1.10 (0.71–1.86)	p=0.698
CIT (per 1 hour)	1.07 (0.91–1.26)	p=0.434	1.06 (0.91–1.25)	p=0.438

Firth estimates with 95% CI and p-values by profile penalized likelihood. Maximum-likelihood CIs are Wald intervals. n=134; 13 DGF events.

Table S4. Characteristics of recipients with and without Cr and eGFR measurement at 24 months.

	**With 24-month data (n=72)**	**Without 24-month data (n=62)**	**p-value**
**Transplant year**			
2022	33 (45.8)	3 (4.8)	**p<0.001**
2023	39 (54.2)	3 (4.8)	**p<0.001**
2024	0 (0.0)	56 (90.3)	**p<0.001**
**Donor and graft**			
DCD donor	30 (41.7)	29 (46.8)	p=0.603
ECD	47 (65.3)	39 (62.9)	p=0.857
Donor DM	10 (13.9)	13 (21.0)	p=0.359
Donor age (years)	59 (50–66)	60 (53–69)	p=0.352
End-ischemic HMP	7 (9.7)	8 (12.9)	p=0.593
**Recipient**			
Recipient age (years)	58 (50–67)	60 (48–69)	p=0.808
Pre-KT Hb (g/dL)	11.9 (10.9–13.0)	12.1 (11.2–13.3)	p=0.279
DGF	9 (12.5)	4 (6.5)	p=0.381
eGFR at 12 months (mL/min/1.73m^2)	48 (37–59)	49 (40–63)	p=0.378
Cr at 12 months (mg/dL)	1.50 (1.20–1.73)	1.42 (1.18–1.66)	p=0.309

Values are n (%) or median (IQR); Fisher's exact test and Mann–Whitney U test. Of the 62 recipients without 24-month data, 47 had not reached 24 months of follow-up by the data cut-off (February 2026), 3 had died and 12 had their last available visit before 24 months.

Table S5. Univariable logistic regression for DGF: odds ratios per clinical unit and per 1-SD increase.

	OR per clinical unit (95% CI)	p-value	OR per 1-SD increase (95% CI)	SD
**Donor factors**				
Donor type (DCD vs DBD)	0.35 (0.09–1.33)	p=0.122	—	—
Male sex	2.60 (0.68–9.92)	p=0.162	—	—
Age (per 10 years)	1.39 (0.85–2.27)	p=0.187	1.59 (0.80–3.19)	14.14
BMI (per 1 kg/m²)	1.01 (0.90–1.13)	p=0.851	1.06 (0.60–1.87)	5.08
Hypertension	0.95 (0.29–3.08)	p=0.933	—	—
DM	**5.24** (1.57–17.50)	**p=0.007**	—	—
Tobacco	1.33 (0.39–4.58)	p=0.649	—	—
Cr (per 0.1 mg/dL)	1.07 (0.91–1.27)	p=0.409	1.23 (0.75–2.02)	0.29
eGFR (per 10 mL/min/1.73m²)	0.90 (0.69–1.16)	p=0.414	0.80 (0.47–1.37)	20.63
CIT (per 1 hour)	1.07 (0.92–1.23)	p=0.381	1.29 (0.73–2.28)	3.95
HMP time (per 1 hour)	1.13 (0.88–1.46)	p=0.338	1.66 (0.59–4.70)	4.12
**Recipient factors**				
Age (per 10 years)	1.05 (0.71–1.56)	p=0.809	1.08 (0.60–1.93)	14.77
Male sex	1.33 (0.39–4.58)	p=0.649	—	—
BMI (per 1 kg/m²)	0.95 (0.83–1.08)	p=0.431	0.79 (0.44–1.43)	4.47
Hypertension	1.08 (0.13–9.19)	p=0.943	—	—
DM	2.19 (0.69–7.00)	p=0.185	—	—
Tobacco	1.33 (0.41–4.31)	p=0.631	—	—
Time on RRT (per 12 months)	1.27 (0.95–1.70)	p=0.100	1.46 (0.93–2.30)	18.87
Hb (per 1 g/dL)	**0.57** (0.38–0.86)	**p=0.007**	**0.40** (0.21–0.78)	1.60
Operative time (per 30 minutes)	1.15 (0.85–1.56)	p=0.359	1.28 (0.76–2.17)	52.09

HMP time available in 55 grafts and time on dialysis in 126 recipients (pre-emptive transplants excluded); all other variables n=134. p-values are identical for both scalings, as the OR per 1-SD increase is a rescaling of the same coefficient.

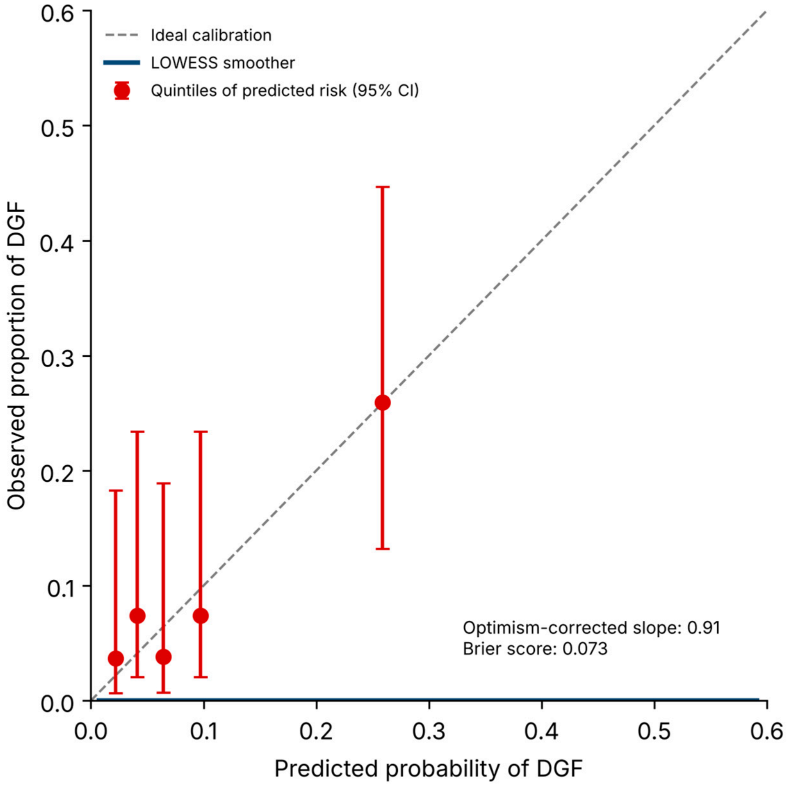

Figure S1. Calibration plot of the multivariable model for DGF (donor DM and pre-KT Hb). The x-axis shows the probability of DGF predicted by the model and the y-axis the proportion of recipients who actually developed DGF. Red dots represent the observed proportion of DGF in each quintile of predicted risk (approximately 27 recipients per quintile), plotted against the mean predicted probability of that quintile, with Wilson 95% confidence intervals; the solid blue line is a LOWESS (locally weighted scatterplot smoothing) curve, which estimates the observed risk of DGF continuously across the range of predicted probabilities by fitting weighted local regressions to the individual observations, without grouping recipients; the dashed diagonal line represents perfect calibration (predicted = observed). Points close to the diagonal indicate good agreement between predicted and observed risk. The bootstrap optimism-corrected calibration slope was 0.91 (ideal value, 1) and the Brier score was 0.073 (0.088 for a null model). Because only 13 DGF events occurred, with 1–2 events in each of the four lower-risk quintiles, the confidence intervals are wide and calibration should be interpreted with caution.
